# A Generic Model for Follicular Lymphoma: Predicting Cost, Life Expectancy, and Quality-Adjusted-Life-Year Using UK Population–Based Observational Data^☆^

**DOI:** 10.1016/j.jval.2018.03.007

**Published:** 2018-10

**Authors:** Han-I Wang, Eve Roman, Simon Crouch, Eline Aas, Cathy Burton, Russell Patmore, Alexandra Smith

**Affiliations:** 1 Epidemiology & Cancer Statistics Group (ECSG), Department of Health Sciences, University of York, York, UK; 2 Department of Health Management and Health Economics, University of Oslo; Oslo, Norway; 3 Haematological Malignancy Diagnostic Service, St. James’s University Hospital, Leeds, UK; 4 Queen’s Centre for Oncology and Haematology, Castle Hill Hospital, Hull, UK

**Keywords:** cost, cost-effectiveness analysis, discrete event simulation, economic evaluation, follicular lymphoma, patient level simulation

## Abstract

**Objectives:**

To use real-world data to develop a flexible generic decision model to predict cost, life expectancy, and quality-adjusted life-years (QALYs) for follicular lymphoma (FL) in the general patient population.

**Methods:**

All patients newly diagnosed with FL in the UK’s population-based Haematological Malignancy Research Network (www.hmrn.org) between 2004 and 2011 were followed until 2015 (N = 740). Treatment pathways, QALYs, and costs were incorporated into a discrete event simulation to reflect patient heterogeneity, including age and disease management. Two scenario analyses, based on the latest National Institute for Health and Clinical Excellence (NICE) guidelines (rituximab induction therapy for newly diagnosed asymptomatic patients and rituximab maintenance therapy for patients between treatments), were conducted and their economic impacts were compared to current practice.

**Results:**

Incidence-based analysis revealed expected average lifetime costs ranging from £6,165 [US$7,709] to £63,864 [US$79,862] per patient, and average life expectancy from 75 days to 17.56 years. Prevalence-based analysis estimated average annual treatment costs of £60–65 million [US$75-80 million], accounting for approximately 10% of the United Kingdom’s annual National Health Service budget for hematological cancers as a whole. Assuming that treatment effects reported in trials are applicable to all patient groups, scenario analyses for two recent NICE guidelines demonstrated potential annual cost savings for the United Kingdom that ranged with uptake frequency from £0.6 million to £11 million [US$0.75-2.75 million].

**Conclusions:**

Costs, survival, and QALYs associated with FL vary markedly with patient characteristics and disease management. Allowing the production of more realistic outcomes across the patient population as a whole, our model addresses this heterogeneity and is a useful tool with which to evaluate new technologies/treatments to support healthcare decision makers.

## Introduction

Accounting for approximately 20% of all non-Hodgkin lymphomas (NHLs), approximately 1860 patients are newly diagnosed with follicular lymphoma (FL) each year in the United Kingdom [1], [2], [3]. FL, the most common of the indolent NHLs, typically follows a remitting relapsing course; initial management ranges from “watch-and-wait (W&W)” (active monitoring/observation) to immediate treatment with chemotherapy/radiotherapy or palliative care. In most cases, therapy is given in response to symptoms, with some patients having several lines of treatment while others remain on W&W. More recently, however, instead of simple W&W, the use of rituximab induction therapy as a strategy to delay the need for chemotherapy/radiotherapy has been recommended and adopted as a treatment option in newly diagnosed asymptomatic patients with advanced stage disease [4]. Although FL is currently incurable, the numbers and combinations of life-prolonging treatments (chemotherapies including novel targeted agents and radiotherapy) is expanding; with individual patients differing widely in their need for, and response to, different treatment regimens the resulting patient pathways are becoming increasingly complex and diverse. This heterogeneity, coupled with the fact that FL in approximately 20% of patients transforms to the more aggressive NHL subtype diffuse large B-cell lymphoma (DLBCL), makes decision making on resource allocation challenging.

In recent years, a number of economic studies have been carried out in FL treatment trials [5], [6], [7], [8], [9], [10], [11], [12], [13], [14], [15], [16], [17], [18], [19], [20], [21], [22]. The majority of these have focused on comparing the cost-effectiveness of administering the monoclonal antibody rituximab at particular points along the patient pathway, either in combination with chemotherapy (immunochemotherapy) both as first-line therapy and subsequently for relapsed/refractory disease, or alone (monotherapy) either as frontline in the W&W phase or as maintenance during remission [7], [8], [9], [10], [11], [12], [13], [14], [15], [16], [17], [18], [19], [20], [21], [22]. However, the findings from such studies can provide only limited information to policymakers, not only because they relate to selected patients at specific points in time but also because certain groups, such as those treated palliatively and those whose disease transforms to DLBCL, are excluded [7], [8], [9], [10], [11], [12], [13], [14], [15], [16], [17], [18], [19], [20], [21], [22].

The overarching aims of the present study were twofold: first, to provide insight into real-world FL treatment costs, survival, and quality-adjusted life-years (QALYs), and second to develop a generic FL model that 1) modelled the whole treatment pathway, rather than being limited to a specific treatment line or agent, 2) reflected real world practice rather than the idealized predefined setting of a randomised controlled trial, 3) predicted medical costs, life expectancy and quality-adjusted life years (QALY) throughout the treatment pathway, and 4) allowed different scenarios to be run, in order to evaluate the impact of changes in disease management on both cost and survival.

## Methods

### Data Sources

The individual-level data used for constructing the simulation model are from the United Kingdom’s Haematological Malignancy Research Network (https://www.HMRN.org), a specialist population-based registry that since 2004 has tracked all patients newly diagnosed with hematological cancers (lymphomas, leukemias, and myelomas) in a catchment population of approximately 3.8 million. Details of the methods underpinning HMRN are described elsewhere [1], [23], [24]. Key to the present report is the fact that HMRN has Section 251 support under the NHS Act 2006, which allows full-treatment, response, and outcome data to be collected to clinical trial standards regardless of patient consent, as well as “flagging” for death at the national Medical Research Information Service (MRIS) and linkage to nationwide information on Hospital Episode Statistics (HES).

The current study includes all 740 patients 18 years of age or older newly diagnosed with FL (International Classification of Disease for Oncology, 3rd ed.: 9690/3, 9698/3) between September 1, 2004 and August 31, 2011 within HMRN’s catchment population. For the purposes of the present analyses, all patients were followed until August 31, 2015, death, or disease transformation to the more aggressive DLBCL. Treatment pathways were mapped according to the management/therapies received. A detailed summary of patient characteristics is presented in Supplementary Table 1.

### Model Structure

To reflect real-world treatment strategies, as well as the heterogeneity of patient characteristics, a discrete event simulation (DES) model was constructed using Simul8 software (Simul8 2017 Professional version, Simul8 Corporation, Boston, MA, USA).

**Fig. 1:**
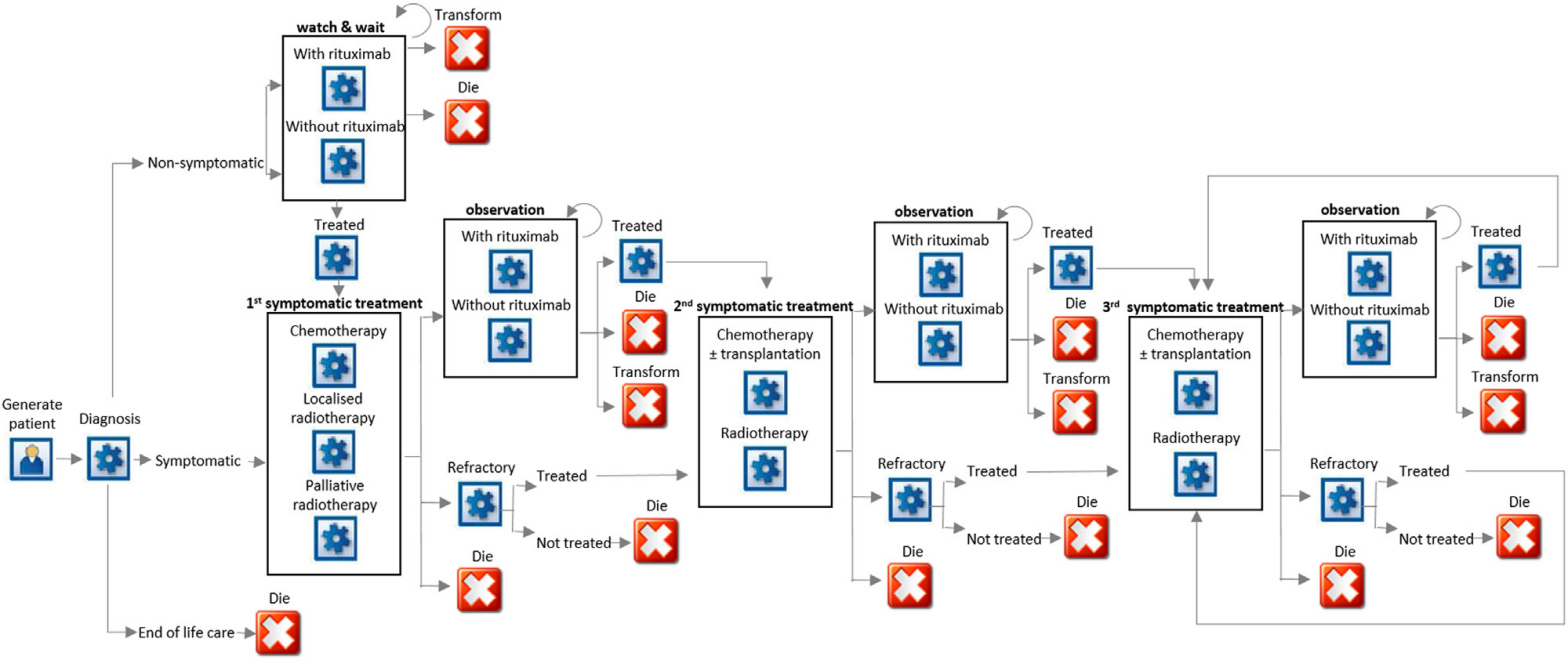
Model structure.

Figure 1 shows the model structure, which is based on real patient pathways, clinical experience (RP, CB), and published clinical guidelines [25]. The key input parameters used in the model are listed in Table 1. For more details, please refer to Supplementary Tables 2 and 3.

**Table 1:** Key parameters

Parameters	Estimates	Distribution	
*Patient generation state*			
Age (years)	Empirical	Weibull	Mean (SD): 64.0 (13.2)
			Weibull (5.57, 69.33)
Age and sex			
Age ≤30	Male: 0.80	Beta	α = 4, β = 1
Age 30–40	Male: 0.60	Beta	α = 15, β = 10
Age 40–50	Male: 0.56	Beta	α = 44, β = 35
Age 50–60	Male: 0.45	Beta	α = 69, β = 86
Age 60–70	Male: 0.44	Beta	α = 95, β = 120
Age 70–80	Male: 0.47	Beta	α = 82, β = 93
Age ≥80	Male: 0.31	Beta	α = 27, β = 59

*Initial treatment decision*			
Initial treatment types			
Age ≤30	Chemotherapy: 0.07	Dirichlet	α_1_ = 2
	Radiotherapy for stage IA: 0.00		α_2_ = 0
	Radiotherapy for IB, II, III, and IV: 0.06		α_3_ = 1
	W&W: 0.87		α_4_ = 2
	Not treated: 0.00		α_5_ = 0
Age 30–40	Chemotherapy: 0.61	Dirichlet	α_1_ = 14
	Radiotherapy for stage IA: 0.01		α_2_ = 2
	Radiotherapy for IB, II, III, and IV: 0.03		α_3_ = 1
	W&W: 0.35		α_4_ = 8
	Not treated: 0.00		α_5_ = 0
Age 40–50	Chemotherapy: 0.40	Dirichlet	α_1_ = 36
	Radiotherapy for stage IA: 0.07		α_2_ = 4
	Radiotherapy for IB, II, III, and IV: 0.08		α_3_ = 6
	W&W: 0.44		α_4_ = 32
	Not treated: 0.01		α_5_ = 1
Age 50–60	Chemotherapy: 0.46	Dirichlet	α_1_ = 73
	Radiotherapy for stage IA: 0.14		α_2_ = 21
	Radiotherapy for IB, II, III, and IV: 0.03		α_3_ = 7
	W&W: 0.37		α_4_ = 54
	Not treated: 0.00		α_5_ = 0
Age 60–70	Chemotherapy: 0.51	Dirichlet	α_1_ = 97
	Radiotherapy for stage IA: 0.11		α_2_ = 22
	Radiotherapy for IB, II, III, and IV: 0.03		α_3_ = 11
	W&W: 0.34		α_4_ = 81
	Not treated: 0.01		α_5_ = 4
Age 70–80	Chemotherapy: 0.46	Dirichlet	α_1_ = 74
	Radiotherapy for stage IA: 0.06		α_2_ = 12
	Radiotherapy for IB, II, III, and IV: 0.03		α_3_ = 10
	W&W: 0.44		α_4_ = 77
	Not treated: 0.01		α_5_ = 2
Age ≥ 80	Chemotherapy: 0.04	Dirichlet	α_1_ = 34
	Radiotherapy for stage IA: 0.06		α_2_ = 2
	Radiotherapy for IB, II, III, and IV: 0.07		α_3_ = 5
	W&W: 0.75		α_4_ = 39
	Not treated: 0.08		α_5_ = 6
W&W decisions (induction rituximab/observation only)			
Age ≤30	Induction rituximab: 0.00	Beta	α = 0, β = 2
Age 30–40	Induction rituximab: 0.00	Beta	α = 0, β = 8
Age 40–50	Induction rituximab: 0.00	Beta	α = 0, β = 32
Age 50–60	Induction rituximab: 0.02	Beta	α = 1, β = 53
Age 60–70	Induction rituximab: 0.03	Beta	α = 3, β = 78
Age 70–80	Induction rituximab: 0.01	Beta	α = 1, β = 76
Age ≥ 80	Induction rituximab: 0.00	Beta	α = 0, β = 39

*Utility*			
Pretreatment	0.83 (SE: 0.06)	Beta	α = 31.7, β = 6.49
Not treated	0.50 (SE: 0.06)	Beta	α = 34.2, β = 34.2
W&W*	0.85 (SE: 0.02)	Beta	α = 270.1, β = 47.66
First-line treatment	0.83 (SE: 0.02)	Beta	α = 291.95, β = 59.8
First remission	0.88 (SE: 0.01)	Beta	α = 928.4, β = 126.6
Subsequent treatment*	0.62 (SE: 0.06)	Beta	α = 161.68, β = 99.1
Subsequent remission*	0.79 (SE: 0.03)	Beta	α = 144.83, β = 38.5

W&W, watch and wait.

* The utilities were derived from Wilder 2006 [39].

The model first assigns attributes: age at diagnosis, sex, disease stage, and prognostic index (FLIPI–FL International Prognostic Index [26]) to a simulated patient group based on HMRN’s study population distributions. Each patient then moves forward to the next event, with probabilities based both on his or her own characteristics and on his or her event history, rather than fixed-time cycles.

The pathways of all simulated patients are modeled starting from the date of diagnosis, with costs of diagnostic biopsies, scans, electrocardiography (ECG), and echocardiography (ECHO) included. Each patient is then assigned to one of three different treatment options determined by his or her baseline characteristics: W&W (with or without rituximab monotherapy), first-line chemotherapy or radiotherapy, or supportive end-of-life care. Patients initially assigned to W&W can go on to receive first-line chemotherapy and/or radiotherapy when their cancer becomes symptomatic, and at this point in the pathway (first-line) radiotherapy can be localized (stage IA disease) or palliative (symptom control). The disease of patients on W&W may also undergo transformation to the more aggressive, but potentially curable, DLBCL; and at this point patients exit the FL model and enter the DLBCL model described in an earlier report [23].

After first-line treatment, one of three events can occur: entry to a period of observation (FL responded to treatment) or, for those who are refractory to treatment, further treatment or death, with the probabilities of these outcomes varying with the type of first-line therapy received, cancer stage, and age at diagnosis. Subsequently, patients on observation can receive second-line treatment because of relapse or exit because of death or transformation to DLBCL. Second-line chemotherapy regimens may differ from first line, and stem cell transplants (SCTs, autologous or allogeneic) may also be given at this point. After second-line treatment, patients may enter a further period of observation and some will go on to receive third-line treatments. In our data, relatively few patients received treatment post third line and, for modeling purposes, it was assumed that the treatment patterns and response rates were similar to those observed at third line.

### Model Inputs

#### Medical costs

The model was built from a NHS perspective; the FL-related medical costs considered those associated with diagnosis, monitoring, treatment, as well as those for supportive and end-of-life care. All treatment details were derived from HMRN’s population-based cohort, and all resource usage, including outpatient visits, day case, inpatient stays, intensive care, and managing treatment side effects/complications (when the episode was related to FL; International Classification of Diseases 10th Revision [ICD 10] code C82) were derived from HES data. Details of the cost items and different chemotherapy regimens included in each costing phase are in Supplementary Table 4.

The cost information (unit costs) used in the model are summarized in Table 2 and are expressed in 2016 UK sterling and 2016 US dollar (£1 = US$1.2505). Parameters were obtained from three sources: the National Tariff 2016/17 for reimbursement/expenditure of NHS treatments [27], Leeds Teaching Hospital NHS Trust for chemotherapy costs that are locally negotiated, and the inflated NHS reference cost 2015/16 when other sources were not available [28].

**Table 2:** Summary of key unit costs

	Unit cost	Source
*Inpatient stay*		
Spell cost	£759	National Tariff
Cost per excess bed day	£232	National Tariff
*Outpatient visit*		
First attendance (single professional)	£288	National Tariff
First attendance (multiple professional)	£463	National Tariff
Follow–up visit (single professional)	£120	National Tariff
Follow–up visit (multiple professional)	£216	National Tariff
*Radiotherapy*		
Planning	£769	National Tariff
Per fraction	£120	National Tariff
*Chemotherapy (per cycle)*		
CVP	£300	Leeds Teaching Hospital Trust
R-CVP	£1,560	Leeds Teaching Hospital Trust
CHOP	£303	Leeds Teaching Hospital Trust
R-CHOP	£1,817	Leeds Teaching Hospital Trust
Chlorambucil	£102	Leeds Teaching Hospital Trust
R-Chlorambucil	£1,867	Leeds Teaching Hospital Trust
Bendamustine	£5,089	Leeds Teaching Hospital Trust
R-Bendamustine	£6,855	Leeds Teaching Hospital Trust
DHAP	£609	Leeds Teaching Hospital Trust
R-DHAP	£2,050	Leeds Teaching Hospital Trust
R-ESHAP	£3,511	Leeds Teaching Hospital Trust
*Transplant*		
Autograft bone marrow transplant	£5,786	Reference cost
Allogeneic bone marrow transplant	£46,608	Reference cost

R-Bendamustine, bendamustine, and rituximab; R-Chlorambucil, chlorambucil, and rituximab; CHOP, cyclophosphamide, doxorubicin, vincristine, and prednisone; R-CHOP, cyclophosphamide, doxorubicin, vincristine, prednisone, and rituximab; CVP, cyclophosphamide, vincristine, and prednisone; R–CVP, cyclophosphamide, vincristine, prednisone, and rituximab; DHAP, dexamethasone, cytarabine, and cisplatin; R–DHAP, dexamethasone, cytarabine, cisplatin, and rituximab; R–ESHAP, etoposide, methylprednisolone, cytarabine, cisplatin, and rituximab.

#### Time-to-event

**Fig. 2:**
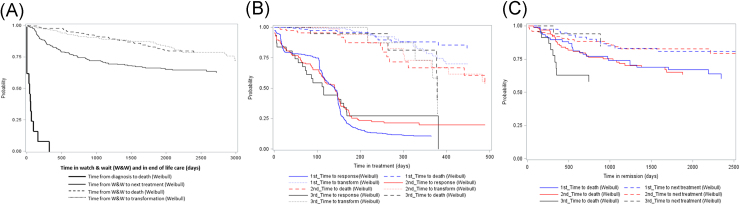
Time-to-event analyses. (A) Time in W&W and in end-of-life care. (B) Time from treatment to death, transformation, or response. (C) Time from response to death or next treatment.

To construct the discrete event simulation model, several time-to-event (TTE) analyses were performed using empirical data to estimate the distributions associated with time between two events. This included the time from diagnosis to treatment, duration of treatment, time from response to next treatment, time from response to death, time from response to disease transformation, and time in end-of-life care. To extrapolate beyond the end of follow-up (August 2015), parametric survival analyses were performed based on best-fit distributions as a function of the patient’s age, treatment details, and response. Six parametric distributions (normal, exponential, Weibull, gamma, log-normal, and log-logistic distributions) were tested, and the best fit model was determined using Akaike Information Criteria (AIC) score and Bayesian information criteria (BIC) [29]. The observed and best fit Kaplan–Meier time to event curves used in the model are shown in Figure 2. More details are provided in Supplementary Table 5. To allow study subjects to proceed from one event to the next, the times to the next possible event are sampled and compared, and the event that occurs first is taken as the next event. With this mechanism, we were able to model all the patients in the study through their treatment pathways from diagnosis to death.

#### Utility

The utility values incorporated into the model were derived directly from a subgroup study of 181 patients with FL diagnosed between 2012 and 2016, all of whom completed EQ-5D-5L questionnaires around the time of diagnosis, and then at 6 to 12 months thereafter until the end of follow-up (January 1, 2017). Information from the published literature was used when the number of patients in a health phase was too small for analysis (fewer than five). A summary of the utilities in each health phase is provided in Table 1.

### Model Outputs

Health outcome was measured by life-years (LYs) and QALYs, while economic outcome was captured by treatment costs. Both economic and health outcomes were discounted using a 3.5% annual discount rate, based on UK guidance recommended by the National Institute for Health and Clinical Excellence (NICE) [30].

### Assessing Uncertainty

Probabilistic sensitivity analysis was performed on all parameters to explore the cumulative uncertainty of the model and possible/likely outcomes. Each parameter was assigned a distribution to reflect sample variability (e.g., beta distributions were used for binominal events), while coefficients of survival models were assigned multivariate normal distributions using the variance and covariance matrix of the coefficients. Then, Monte Carlo simulations were performed by sampling parameters from the corresponding distributions simultaneously 1000 times; stable results occurred in this study before 1000 was reached. All outputs from the iterations were summarized with 95% confidence intervals [31].

### Analysis

#### Base case

To investigate the economic impact of FL across the United Kingdom as a whole, the annual number of expected cases (N = 1860) estimated by HMRN (https://www.hmrn.org/statistics/incidence) was used in the model. To explore the effects of the time horizon on cost, QALYs, and survival, results are presented in aggregate and for the time horizons of 5 years, 10 years, 15 years, 20 years, 25 years, 30 years, and lifetime (simulated until 100 years of age or death). The model further generated prevalence-based estimates, calculating the annual impacts on cost, Lys, and QALYs for treating new and existing FL patients in the United Kingdom. The rate of entry into the model was determined by the incidence rate, and results were collected after a burn-in period of 30 years.

#### Scenarios

To explore the impact of current and future policy changes, the model was used in two scenarios. Scenario 1 was based on recent UK NICE guidelines [32]: Patients with FL who are not symptomatic at diagnosis can be given rituximab once a week for 4 weeks as part of the W&W management strategy. To evaluate the economic impact of this change, we modeled uptake frequencies of 25%, 50%, 75%, and 100% and combined the costs from our observational data with the efficacy findings from the trial [4], which reported that monotherapy rituximab significantly delayed the need for subsequent chemotherapy/radiotherapy treatment (hazard ratio [HR]: 0.35, 95% CI: 0.22–0.56). Scenario 2 was based on another NICE appraisal [33]: FL patients who respond to treatment may also be given rituximab once every 2 months for a maximum of 2 years. To estimate the economic impact of this change, we again modeled uptake frequencies of 25%, 50%, 75%, and 100% and used the costs from our observational data and the findings from the trial, in which rituximab was reported to have delayed the need for second-line treatment (HR: 0.55, 95% CI: 0.44–0.68) [34], to model efficacy.

### Validation

The model was validated using standard methods, including face, as well as internal and external validations [35]. Face validation on model structure and data sources was conducted while the model was under construction by consulting clinical experts. Internal validation was assessed by comparing predicted costs and LYs with empirical estimates, and external validation was performed by comparing simulated results with the findings from the relevant literature.

## Results

### Incidence-Based Results

Predicted lifetime costs, LYs, and QALYs are presented in Table 3; the detailed predicted resource use (quantity) can be found in Supplementary Table 6. Overall, the average cost per patient was around £18,705 [US$23,390], excluding the impact of transformation to DLBCL. As expected, costs for patients who received treatment with curative intent were higher (£24,872 [US$31,102]) than for those who did not. The highest lifetime costs occurred among patients who received second-line treatment that included an SCT (£60,261 [US$75,356]), but this was accompanied by the longest survival (15.79 LYs). Those who were asymptomatic and remained on W&W throughout incurred the lowest costs (£5,296 [US$6,622]), but had LYs (8.22 LYs) similar to those who were treated (9.72 LYs).

**Table 3:** Simulated medical costs, LYs, and QALYs across treatment pathway over a life–time horizon for FL (N = 1860)

		Incidence–based results					
		Total (FL costs alone)			Total FL + DLBCL		Prevalence–based results
		Cost (£)	LYs	QALYs	Cost (£)	LYs	Annual cost
	**N**	mean (95% CI)	mean (95% CI)	mean (95%CI)	mean (95% CI)	mean (95% CI)	Million (£)
Total	1860	18,705 (18,631–18,781)	9.08 (9.06–9.11)	7.35 (7.34–7.37)	23,122 (23,042–23,201)	11.41 (11.38–11.43)	61.6 (59.2–64.0)
W&W only	550 (548–551)	5,296 (5,290–5,301)	8.22 (8.20–8.24)	7.40 (7.38–7.41)	11,818 (11,794–11,842)	11.61 (11.58–11.63)	18.2 (17.4–18.9)
Treated	1,273 (1,271–1,274)	24,872 (24,765–24,979)	9.72 (9.69–9.75)	8.46 (8.43–8.48)	28,509 (28,394–28,624)	11.65 (11.62–11.68)	42.1 (40.4–43.8)
First line only	720 (717–722)	13,456 (13,388–13,525)	8.27 (8.24–8.31)	8.07 (8.06–8.14)	16,658 (16,581–16,735)	10.01 (9.98–10.05)	23.8 (22.8–24.8)
Second line plus							
With SCT	77 (76–78)	60,261 (59,791–60,730)	15.79 (15.70–15.87)	12.15 (12.09–12.21)	63,864 (63,376–64,351)	17.56 (17.47–17.64)	2.5 (2.4–2.7)
Without SCT	499 (497–502)	36,000 (35,828–36,171)	10.85 (10.81–10.90)	8.34 (8.30–8.38)	40,123 (39,936–40,309)	12.99 (12.94–13.05)	16.5 (15.8–17.2)
Not treated	37 (36–38)	6,165 (6,093–6,237)	0.21 (0.20–0.21)	0.12 (0.12–0.12)	6,165 (6,093–6,237)	0.21 (0.20–0.21)	1.2 (1.1–1.3)

95% CI, 95% confidence interval; DLBCL, diffuse large B-cell lymphoma; FL, follicular lymphoma; LY, life-year; QALY, quality-adjusted life-year; SCT, stem cell transplant (including allograft/autologous SCT); W&W, watch and wait.

To include the impact of transformation to DLBCL on costs and LYs, the model further simulated patients beyond transformation to DLBCL, using previous results [25]. As shown in Table 3, the average cost and LY per patient were £23,122 [US$28,914] and 11.41 years, respectively. Compared to the results that included FL-related elements alone, the differences were considerable (around £4,400 [US$5,502] and 2.33 LYs).

**Fig. 3:**
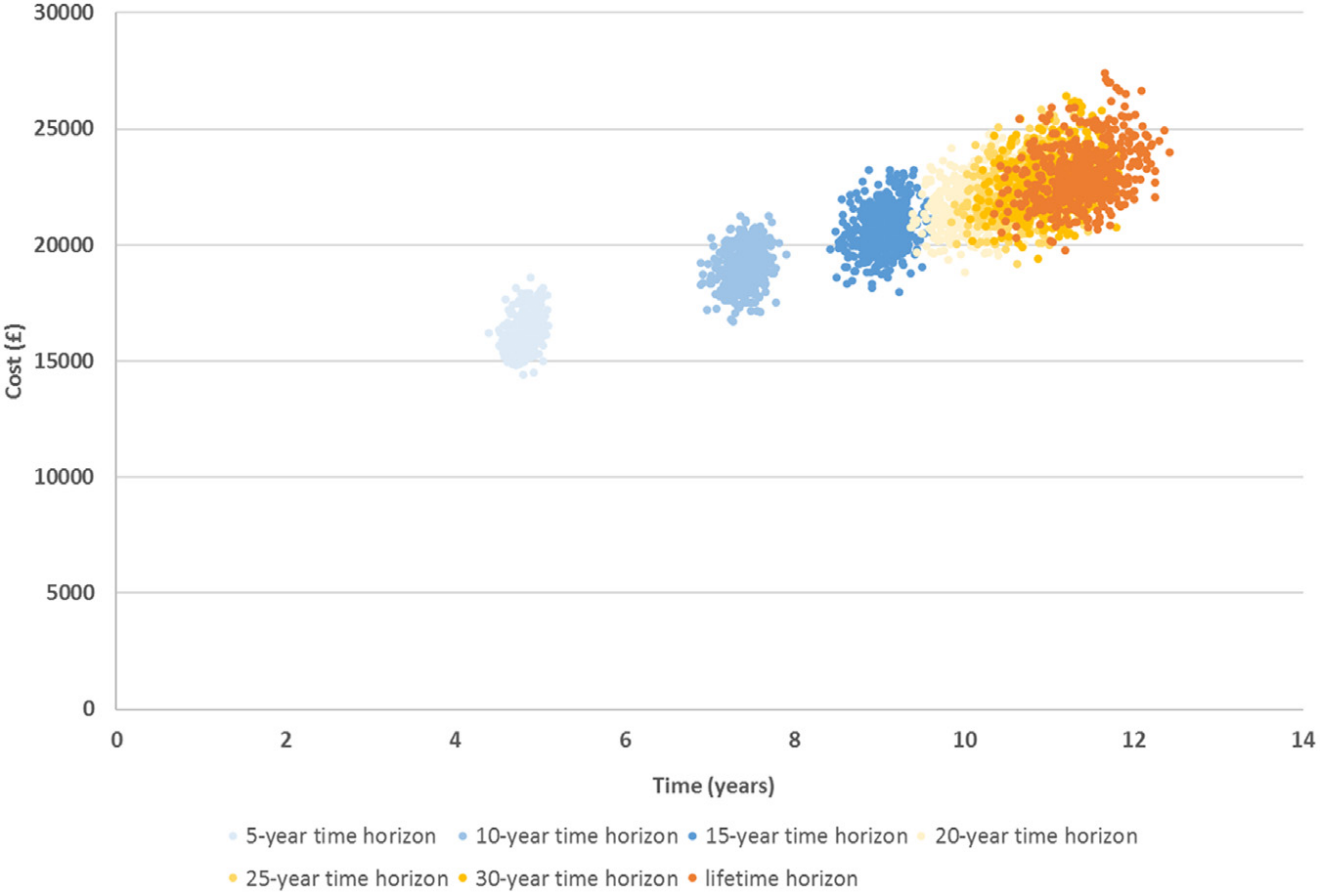
Estimated average costs and LYs per patient over different time horizons (1000 iterations).

Predicted average costs and LYs for 1000 iterations over different time horizons are presented in Figure 3. Results for different time horizons provide insight into the overall economic and health impact across the treatment pathway; results for shorter periods (5-year, 10-year, 15-year, 20-year, 25-year, and 30-year) allowed external validation and exploration of time horizon impact. As shown, the average costs (including treatment following transformation) per patient were £21,761 [US$27,212], £22,399 [US$28,010], £22,718 [US$28,409], and £23,121 [US$28,913] for the 20-year, 25-year, 30-year, and lifetime horizons respectively. The cost continued to increase throughout the time horizons, as did the predicted LYs (10.1, 10.8, 11.0, and 11.4 for 20-year, 25-year, 30-year, and lifetime horizons, respectively).

### Prevalence-Based Results

Prevalence-based costs provide estimates of the total cost associated with treating existing and new FL patients in a 1-year period. The simulation results demonstrate that the annual cost for treating FL across the United Kingdom as a whole was in the region of £50 million [US$62.5 million] for the lower bound and £60 million [US$75 million] for the upper bound (data not shown); the annual cost including treatment following transformation to DLBCL was in the region of £60 million to £65 million [US$75-80 million] (Table 3).

### Scenario Analyses

Extrapolating the effect size shown in the trial (HR point estimate 0.35) to the general patient population, Table 4 demonstrates the economic impact of introducing first-line rituximab to patients who are currently managed on W&W without rituximab monotherapy. As shown, the estimated annual cost of treating new and existing FL patients would decrease by approximately £2.4 million to £3.6 million [US$3.0-4.5 million] if uptake was 100%, £1.5 million to £2.4 million [US$1.9-3.0 million] for 75%, £1.4 million to £1.9 million [US$1.8-2.4 million] for 50%, and £0.1 million to £2.1 million [US$0.1-2.6 million] for 25% (including costs associated with transformation to DLBCL).

**Table 4:** Scenario analysis results based on uptake frequencies and treatment effects

Uptake frequency	Scenario 1: Rituximab during W&W*	Scenario 2: Rituximab during observation†
	Mean cost (min–max) £	Mean cost (min–max) £
Base case‡	61.6 Million (59.2–64.0 million)	61.6 Million (59.2–64.0 million)
25%	60.5 Million (57.1–63.9 million)	58.6 Million (57.1–60.4 million)
50%	60.3 Million (57.8–62.1 million)	55.9 Million (53.0–57.9 million)
75%	59.5 Million (57.7–61.6 million)	54.2 Million (52.6–55.7 million)
100%	58.7 Million (56.8–60.4 million)	52.3 Million (50.3–53.6 million)

W&W, watch and wait.

* Based on the trial effect size of hazard ratio 0.35; 95% confidence intervals 0.22–0.56 [4].

† Based on the trial effect size of hazard ratio 0.55; 95% confidence intervals (0.44–0.68) [33].

‡ Current study; 2% of patients received rituximab during W&W, and 15% during subsequent periods of observation/maintenance.

Likewise, Table 4 shows the economic impact of introducing rituximab to patients in remission, assuming the trial effect size can be extrapolated to the patient population as a whole (HR point estimate 0.55). Despite extra spending on the cost of maintenance rituximab, the estimated annual costs would decrease by around £8.9 million to £10.4 million [US$75-80 million] when uptake rate is 100%, £6.6 million to £8.3 million [US$8.3-10.4 million] for 75%, £5.7 million to £6.1 million [US$7.1-7.6 million] for 50%, and £3.0 million to £3.6 million [US$3.7-4.5 million] for 25% when transformation to DLBCL is taken into consideration.

### Model Validation

**Fig. 4:**
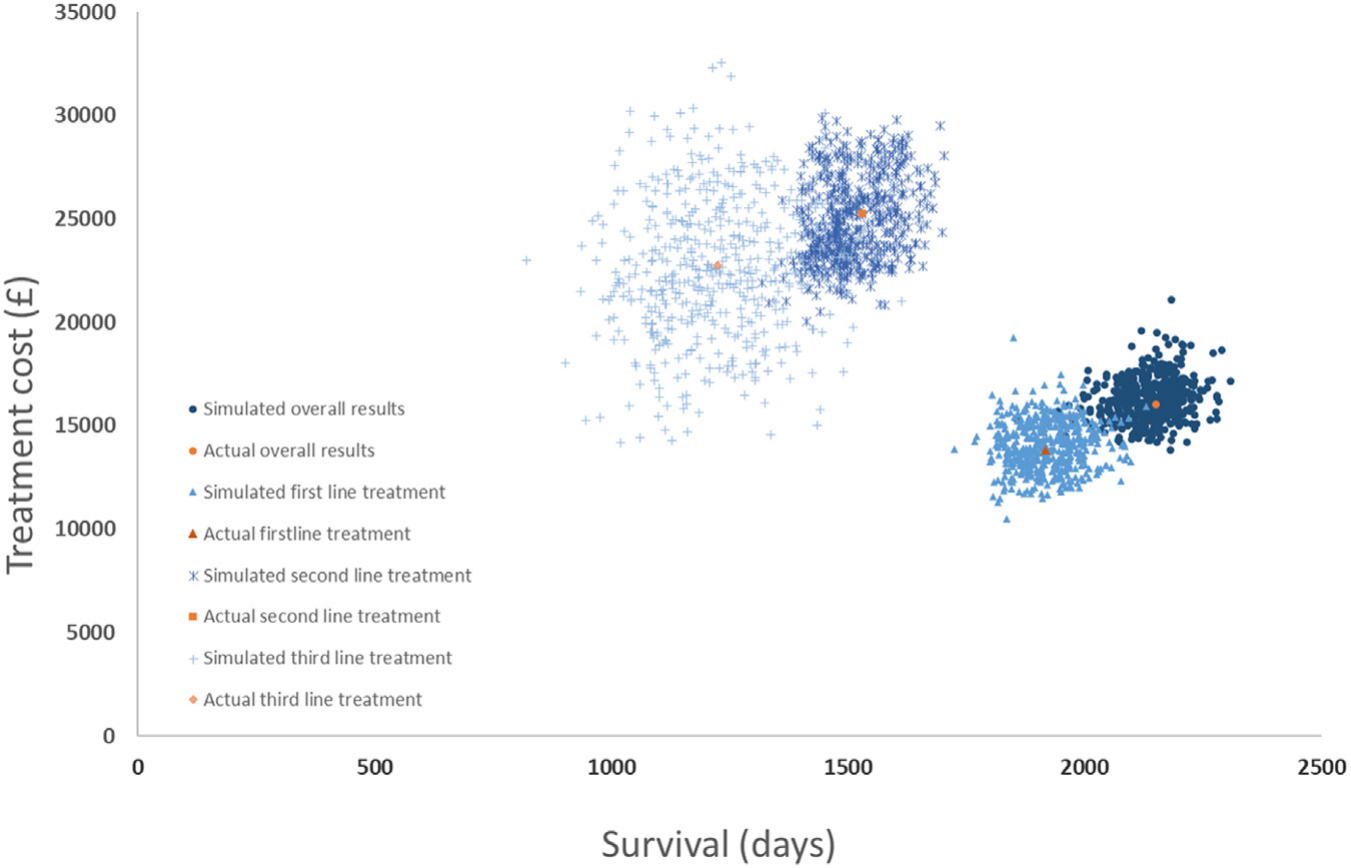
Model validation.

Regarding face validity, the model structure, parameters, and results were checked and approved by the clinical authors (RP, CB). For internal validity, both simulated cost and survival outcomes were compared to empirical estimates derived from the study population (HMRN). The model was simulated to mirror that of the study population, allowing patients to enter the model for 7 years (2004–11) at a constant rate until 740 was reached, and then further modeling for another 4 years. The simulated time to event data closely matched the observed data (the Kaplan–Meier curves are compared in Supplementary Fig. 1). The results from 1000 iterations produced average treatment costs and survival days for all patients (Fig. 4) that were similar to the empirical data. The average duration of disease (from date of diagnosis to either death or modelling end date) was 2378 days per patient (ranging from 2142 to 2608 days across 1000 iterations) and captured 99% of the actual observed time (2395 days). The average simulated cost was £17,331 [US$21,672] per patient (ranging from £14,746 [US$18,440] to £20,556 [US$25,705] across 1000 iterations), which slightly overestimated the empirical results derived from the study population (£17,052 [US$21,324]). The same pattern was observed in the simulation results for each line of treatment (Fig. 4).

With regard to external validity, the simulated cost and survival results were broadly similar to findings reported in the relevant literature (see Supplementary Table 7).

## Discussion

This is the first FL model to simulate individual patients’ medical costs, LYs, and QALYs along the full patient pathway. The analysis revealed that although FL is a comparatively rare cancer (annual incidence rate is 3.3 per 100,000) and the average treatment cost per patient is relatively modest (£23,122 [US$28,914]), the economic impact of treating new and existing FL patients is significant; the annual cost in the United Kingdom lies in the region of £60 million to £65 million [US$75-80 million], accounting for approximately 10% of the annual NHS budget for hematological cancers as a whole [36]. Our analysis also confirmed the underlying heterogeneity of the FL patient population; the expected lifetime medical cost ranged from £6,165 [US$7,709] to £63,864 [US$79,862] per patient and life expectancy from 75 days to 17.56 years (see Table 3 and Supplementary Table 8 for more details). In this context, the application of “averages” may not be appropriate in all situations.

The importance of adopting a lifetime horizon approach was also demonstrated in the current study. The differences in estimated costs derived from 20-year, 25-year, and 30-year horizons were relatively minor, reflecting the fact that the majority of the treatment occurred in the first 20 years. However, the differences in estimated LYs were considerable, confirming, as has been suggested by others, that the lifetime horizon is the optimal approach as it allows the overall effect of treatment to be fully captured. As such, results from studies using time horizons of less than 20 years need to be interpreted cautiously, as the results are unlikely to reflect true differences in LYs.

To demonstrate the flexibility/adaptability of the generic model developed in the current study, we tested two scenarios based on NICE guidelines and explored their annual economic impacts on the health care system using effect sizes reported in clinical trials [4], [34], which may or may not be generalizable to the patient population as a whole. In this context, the model performed well, producing results that are broadly consistent with the findings from relevant trial-based literature, although the costs for both scenarios were on the lower side [10], [11], [14], [16], [18], [20], [21], [22]. In addition, although both scenarios demonstrated expected savings, wider dispersion of annual economic impact was observed in Scenario 2. This could possibly be because cost savings are driven not only by the reported treatment effects and uptake frequencies, but also by the number of patients who will benefit and whether the need for subsequent treatment can be prevented. Furthermore, although potential cost savings are confirmed, such estimates need to be interpreted cautiously, as they are applicable only in situations in which treatment effects derived from trial populations extrapolate to the general patient population. Importantly, however, the model has the ability to accommodate heterogeneity and, as new data emerge, the estimates can be refined to more accurately reflect the real-world impact. In addition, the generic approach could aid future cost-effectiveness studies examining new interventions, including, for example, the introduction of biosimilars for rituximab, the usage of which is increasing now that the original patent has expired [37], [38].

The ability to model patient pathways in a more granular fashion than is usually the case is a major strength of our approach. This more realistic structure allows the model to more closely mirror clinical practice, enabling differences in cost and health outcomes to be characterized more effectively. The incorporation of real-world QALY information, rather than hypothetical figures, also means that more reliable QALY results relating to FL treatment can be predicted. Another strength of this study is the flexibility/adaptability of the model, which allows parameters to be varied and different time horizons to be adopted, permitting the model to evaluate different types of intervention, health care settings, and diseases. A tutorial video demonstrating the flexibility of the interactive model can be found at https://www.hmrn.org/economics/models. In addition, the model can simulate both open and closed cohorts to answer prevalence- and incidence-based questions. Furthermore, the model has the potential and flexibility to evaluate treatment sequences, although such analysis is currently outside the scope of the present study. It is important to note, however, that others have used similar modeling techniques to explore the optimal treatment sequence for the management of conditions where options change as the disease progresses [15], [39], [40], [41], [42]. Like our approach, these studies modeled patients through their pathways; the main difference was that they simulated a set of key treatment sequences one at a time, rather than all treatment pathways/sequences at the same time [15], [39], [40], [41], [42]. The main benefit of modeling all treatment pathways at the same time is the ability to demonstrate impacts in the real-world setting, hence allowing policymakers to make more informed decisions.

Our study is subject to some limitations, primarily those relating to the extrapolation of data. For example, we used parametric disease-free techniques to extrapolate survival beyond the 10-year follow-up period, having confirmed that the observed survival differed from that reported in UK life tables. Likewise, data relating to QALYs were derived from a subgroup of patients who could potentially differ in important characteristics from the patient population as a whole (the impact of utilities on QALY estimates is presented in Supplementary Fig. 2). Furthermore, because this is an observational study, it is difficult to compare the efficacy of different regimens, as treatment is not randomly assigned. Finally, as HMRN is an ongoing cohort with continued ascertainment and follow-up, although it was not possible in the present report to estimate utilities by chemotherapy regimen owing to small numbers, more empirical data will be available in time, allowing further refinement of the results.

## Conclusion

The analyses presented here confirm that costs, survival, and QALYs associated with FL vary markedly with patient characteristics and disease management. Based on real-world patient level data, our model addresses this heterogeneity, allowing the production of more realistic outcomes across the patient population as a whole. Future application of the model will include evaluation of new technologies/treatments to support health care decision makers, especially in the era of personalized medicine.
