# Supplementary material for: A Generic Model for Follicular Lymphoma: Predicting Cost, Life Expectancy, and Quality-Adjusted-Life-Year Using UK Population–Based Observational Data
Source: Value Health. 2018 Oct;21(10):1176–85. doi: 10.1016/j.jval.2018.03.007 (PMC6191529; doi:10.1016/j.jval.2018.03.007)
Supplement: Supplementary file 1 — Supplementary material [file mmc1.docx]

**Supplementary Table 1: Baseline characteristics of study population (n=740)**

|  | **Overall** | |  | **Treated** | |  | **Not treated** | |  |
| --- | --- | --- | --- | --- | --- | --- | --- | --- | --- |
|  | **N** | **%** |  | **N** | **%** |  | **N** | **%** |  |
| Total | 740 | 100% |  | 727 | 98.2% |  | 13 | 1.8% |  |
| Age at diagnosis (years) |  |  |  |  |  |  |  |  |  |
| Median (Q1-Q3) | 64 | (55-73) |  | 63 | (55-73) |  | 74 | (68-81) |  |
| Sex |  |  |  |  |  |  |  |  |  |
| Male | 336 | 45.4% |  | 329 | 45.3% |  | 7 | 53.8% |  |
| Female | 404 | 54.6% |  | 398 | 54.7% |  | 6 | 45.2% |  |
| Age (years) >60 |  |  |  |  |  |  |  |  |  |
| Yes | 476 | 64.3% |  | 464 | 63.8% |  | 12 | 92.3% |  |
| No | 264 | 35.7% |  | 263 | 36.2% |  | 1 | 7.7% |  |
| Stage |  |  |  |  |  |  |  |  |  |
| I | 109 | 14.7% |  | 109 | 15.0% |  | - | - |  |
| II | 94 | 12.7% |  | 94 | 12.9% |  | - | - |  |
| III | 122 | 16.5% |  | 121 | 16.6% |  | 1 | 7.7% |  |
| IV | 328 | 44.3% |  | 321 | 44.2% |  | 7 | 53.8% |  |
| Not fully staged | 87 | 11.8% |  | 82 | 11.3% |  | 5 | 38.5% |  |
| Extranodal Site >1 |  |  |  |  |  |  |  |  |  |
| Yes | 148 | 20.0% |  | 147 | 20.2% |  | 1 | 7.7% |  |
| No | 487 | 65.8% |  | 484 | 66.6% |  | 3 | 23.1% |  |
| Not fully staged | 105 | 14.2% |  | 96 | 13.2% |  | 9 | 69.2% |  |
| Performance status (ECOG) |  |  |  |  |  |  |  |  |  |
| 0 | 407 | 55.0% |  | 407 | 56.0% |  | - | - |  |
| 1 | 260 | 35.1% |  | 255 | 35.1% |  | 5 | 38.5% |  |
| >1 | 73 | 9.9% |  | 65 | 8.9% |  | 6 | 61.5% |  |
| Elevated Lactose Dehydrogenase (LDH) |  |  |  |  |  |  |  |  |  |
| Yes | 193 | 26.1% |  | 189 | 26.0% |  | 4 | 30.8% |  |
| No | 469 | 63.4% |  | 466 | 64.1% |  | 3 | 23.1% |  |
| Not measured | 78 | 10.5% |  | 72 | 9.9% |  | 6 | 46.1% |  |
| Follicular Lymphoma International Prognostic Index (FLIPI) |  |  |  |  |  |  |  |  |  |
| Low (0-1 risk factor) | 192 | 25.9% |  | 192 | 26.4% |  | - | - |  |
| Intermediate (2 risk factors) | 172 | 23.2% |  | 172 | 23.6% |  | - | - |  |
| High (≥ 3 risk factors) | 272 | 36.8% |  | 263 | 36.2% |  | 9 | 69.2% |  |
| Not known | 104 | 14.1% |  | 100 | 13.8% |  | 4 | 30.8% |  |
| First line treatment |  |  |  |  |  |  |  |  |  |
| Chemotherapy | 330 | 44.6% |  | 330 |  |  | - | - |  |
| Radiotherapy | 63 | 8.5% |  | 63 |  |  | - | - |  |
| Radiotherapy for Stage IA | 41 | 5.5% |  | 41 |  |  | - | - |  |
| Observation | 293 | 39.6% |  | 293 |  |  | - | - |  |
| Not treated | 13 | 1.8% |  | - | - |  | 13 | 100% |  |

**Supplementary Table 2: Parameters for first line treatment**

| **Regimen** | **R-CVP** | **R-CHOP** | **R-Chlorambucil** | **Radiotherapy only** |
| --- | --- | --- | --- | --- |
|  |  |  |  |  |
| Number | 250 | 96 | 52 | 44 |
| Treatment type |  |  |  |  |
| From watch & wait |  |  |  |  |
| % Age ≤ 30 | 100 | 0 | 0 | 0 |
| % Age 30-40 | 76 | 24 | 0 | 0 |
| % Age 40-50 | 83.2 | 16.8 | 0 | 0 |
| % Age 50-60 | 73.8 | 6.5 | 0 | 19.7 |
| % Age 60-70 | 79.8 | 8.7 | 11.6 | 4.55 |
| % Age 70-80 | 79.3 | 2.5 | 18.2 | 0 |
| % Age ≥ 80 | 5.4 | 0 | 94.6 | 0 |
|  |  |  |  |  |
| From diagnosis |  |  |  |  |
| % Age ≤ 30 | 100 | 0 | 0 | - |
| % Age 30-40 | 88.1 | 11.9 | 0 | - |
| % Age 40-50 | 63.9 | 16.4 | 5.56 | - |
| % Age 50-60 | 71.8 | 26.8 | 1.4 | - |
| % Age 60-70 | 51.3 | 34.8 | 13.9 | - |
| % Age 70-80 | 68.6 | 17.2 | 14.2 | - |
| % Age ≥ 80 | 65.5 | 3.7 | 30.8 | - |
|  |  |  |  |  |
| % without rituximab |  |  |  |  |
| From watch & wait | 0 | 7.14 | 83.33 | - |
| From diagnosis | 1.55 | 0 | 97.5 | - |
|  |  |  |  |  |
| % treated as inpatient |  |  |  |  |
| From watch & wait | 6.1 | 26.4 | 0 | 0 |
| From diagnosis | 10.0 | 11.1 | 0 | 0 |
|  |  |  |  |  |
| % treated as day case |  |  |  |  |
| From watch & wait | 77.5 | 37.5 | 0 | 100 |
| From diagnosis | 56.7 | 53.6 | 2.6 | 0 |
|  |  |  |  |  |
| % treated as outpatient |  |  |  |  |
| From watch & wait | 16.4 | 36.1 | 100 | 0 |
| From diagnosis | 33.4 | 35.3 | 97.4 | 0 |
|  |  |  |  |  |

* R-CHOP: cyclophosphamide, doxorubicin, vincristine, prednisolone and rituximab

R-CVP: cyclophosphamide, vincristine, prednisolone + rituximab

R-Chlorambucil: Chlorambucil + rituximab

**Supplementary Table 3: Parameters for second and third line treatments**

| **Regimen** | **R-CHOP** | **R-CVP** | **R-Chlorambucil** | **R-DHAP** | **R-Bendamustine** | **Other**† | **Radiotherapy only** |
| --- | --- | --- | --- | --- | --- | --- | --- |
| **Second line treatment** |  |  |  |  |  |  |  |
| % Age ≤ 30 | 0 | 0 | 0 | 100 | 0 | 0 | 0 |
| % Age 30-40 | 10 | 0 | 0 | 43.9 | 0 | 13.9 | 32.1 |
| % Age 40-50 | 19 | 27 | 0 | 29.6 | 2 | 5 | 19 |
| % Age 50-60 | 25.9 | 35.2 | 5 | 4.4 | 0 | 24.5 | 4.9 |
| % Age 60-70 | 18.1 | 18.2 | 11 | 11.1 | 0 | 20.4 | 31.9 |
| % Age 70-80 | 0.1 | 27.1 | 30.3 | 0 | 3.7 | 2.4 | 40 |
| % Age ≥ 80 | 8.3 | 16.7 | 50 | 0 | 0 | 16.7 | 8.3 |
|  |  |  |  |  |  |  |  |
| % without rituximab | 6.9 | 3.4 | 63.6 | 11.1 | 100 | 24 | - |
|  |  |  |  |  |  |  |  |
| % treated as inpatient | 4.9 | 16.3 | 20 | 85.1 | 0 | 0 | 0 |
| % treated as day case | 69.6 | 71.2 | 0 | 4.5 | 0 | 100 | 100 |
| % treated as outpatient | 25.5 | 12.5 | 80 | 10.4 | 1 | 0 | 0 |
|  |  |  |  |  |  |  |  |
| **Third line treatment** |  |  |  |  |  |  |  |
| % Age ≤ 30 | 0 | 11.6 | 0 | 88.4 | 0 | 0 | 0 |
| % Age 30-40 | 0 | 0.7 | 0 | 99.3 | 0 | 0 | 0 |
| % Age 40-50 | 65.1 | 0 | 0 | 17.9 | 0 | 2.7 | 14.3 |
| % Age 50-60 | 15.6 | 6.6 | 0 | 0 | 0 | 37.5 | 40.3 |
| % Age 60-70 | 11.5 | 7.6 | 0 | 19.5 | 10.4 | 7.9 | 43.2 |
| % Age 70-80 | 4.1 | 6 | 34.2 | 0 | 30.4 | 8.7 | 16.6 |
| % Age ≥ 80 | 0 | 0 | 0 | 0 | 0 | 100 | 0 |
|  |  |  |  |  |  |  |  |
| % without rituximab | 0 | 14.3 | 0 | 20 | 0 | 28.6 | - |
|  |  |  |  |  |  |  |  |
| % treated as inpatient | 4.1 | 6.3 | 0 | 20 | 0 | 0 | 0 |
| % treated as day case | 91.7 | 20.8 | 71.4 | 60 | 0 | 100 | 100 |
| % treated as outpatient | 4.2 | 72.9 | 28.6 | 20 | 100 | 0 | 0 |
|  |  |  |  |  |  |  |  |

†Including FC (Fludarabine and Cyclophosphamide), R-FC (Fludarabine, Cyclophosphamide and rituximab), FMD (Fludarabine, Mitoxantrone and Dexamethasone), R-FMD (Fludarabine, Mitoxantrone, Dexamethasone and rituximab), and R-Zevalin (Zevalin and Rituximab)

**Supplementary Table 4: Cost items**

| **Phases** | **Cost items** |
| --- | --- |
| **First line treatment** | Chemotherapy:  CHOP (-R)  CVP (-R)  Chlorambucil (-R)  Radiotherapy  Supportive care:  G-CSF  Transfusion  Post chemotherapy imaging  Out-patient visits |
| **Second line treatment** | Chemotherapy:  CHOP (-R)  CVP (-R)  Chlorambucil (-R)  Bendamustine (-R)  DHAP (-R)  ESHAP (-R)  Radiotherapy  Supportive care:  G-CSF  Transfusion  Autograft bone marrow transplant  Allogeneic bone marrow transplant  Post chemotherapy imaging  Out-patient visits |
| **Third line treatment** | Chemotherapy:  CHOP (-R)  CVP (-R)  Chlorambucil (-R)  Bendamustine (-R)  DHAP (-R)  ESHAP (-R)  Radiotherapy  Supportive care:  G-CSF  Transfusion  Autograft bone marrow transplant  Allogeneic bone marrow transplant  Post chemotherapy imaging  Out-patient visits |
| **Remission** | Out-patient visits (follow up)  Rituximab (maintenance) |
| **Follow up** | Out-patient visits |
| **End-of-life care** | In-patient treatment  Out-patient visits |
|  |  |

CVP (cyclophosphamide, vincristine and prednisone); R-CVP (cyclophosphamide, vincristine, prednisone and rituximab); CHOP (cyclophosphamide, doxorubicin, vincristine and prednisone); R-CHOP (cyclophosphamide, doxorubicin, vincristine, prednisone and rituximab); R-Chlorambucil (chlorambucil and rituximab); R-Bendamustine (bendamustine and rituximab); DHAP (dexamethasone, cytarabine and cisplatin); R-DHAP (dexamethasone, cytarabine, cisplatin and rituximab); R-ESHAP (etoposide, methylprednisolone, cytarabine, cisplatin and rituximab).

**Supplementary Table 5: Coefficients (95% Confidence Intervals) of the time to event models**

|  | **Diagnosis to next treatment** | **Time from Tx1 to death** | **Time from Tx1 to transform** | **Time from Tx1 to CR1** | **Time from CR1 to death** | **Time from CR1 to 1^st^ relapse** |
| --- | --- | --- | --- | --- | --- | --- |
| **Distribution** | Weibull | Weibull | Weibull | Weibull | Weibull | Weibull |
| **Shape** | 1.10 (1.03-1.16) | 0.98 (0.72-1.33) | 1.89 (1.63-2.17) | 0.75 (0.70-0.80) | 0.99 (0.78-1.25) | 0.75 (0.65-0.87) |
| **Intercept** | 3.48 (3.11-3.84) | 14.98 (10.41-19.55) | 7.48 (6.31-8.66) | 5.29 (4.51-6.08) | 14.80 (12.25-17.34) | 8.36 (7.05-9.67) |
| **Age** | -0.002 (-0.007-0.003) | -0.09 (-0.15- -0.04) | -0.005 (-0.02-0.01) | 0.002 (-0.009-0.013) | -0.08 (-0.11- -0.04) | 0.002 (-0.02-0.02) |
| **FLIPI** |  |  |  |  |  |  |
| **High** | reference | reference | reference | reference | reference | reference |
| **Intermediate** | -0.05 (-0.23-0.13) | 0.45 (-1.14-2.04) | -0.24 (-0.69-0.20) | 0.13 (-0.23-0.48) | 0.13 (-0.71-0.98) | 0.41 (-0.22-1.04) |
| **Low** | 0.13 (-0.06-0.32) | 0.55 (-1.06-2.16) | 0.26 (-0.36-0.88) | 0.18 (-0.23-0.58) | 1.93 (0.45-3.41) | 0.82 (0.10-1.54) |
| **Not known** | -0.08 (-0.29-0.13) | -0.06 (-1.36-1.24) | -0.58 (-1.08- -0.09) | -0.28 (-0.72-0.17) | -0.01 (-0.84-0.82) | 0.16 (-0.59-0.91) |
| **Treatment** |  |  |  |  |  |  |
| **Chemotherapy** | reference | - | - | - | - | - |
| **CHOP** | - | -0.89 (-2.13-0.36) | -0.33 (-0.80-0.14) | -0.28 (-0.62-0.06) | -0.35 (-1.15-0.44) | 0.81 (0.09-1.53) |
| **CVP** | - | reference | reference | reference | reference | reference |
| **Chlorambucil** | - | -0.07 (-1.27-1.13) | 0.36 (-0.10-0.82) | 0.55 (0.07-1.03) | -0.99 (-1.80- -0.18) | -0.83 (-1.54- -0.13) |
| **Radiotherapy for IA** | 0.88 (0.61-1.16) | 22.66 (-1073-1073) | -0.004 (-0.77-0.76) | -1.44 (-1.91- -0.97) | -0.36 (-1.63-0.91) | 0.58 (-0.31-1.47) |
| **Radiotherapy** | 0.90 (0.60-1.20) | -0.65 (-2.84-1.54) | 0.28 (-0.44-1.01) | -0.86 (-1.37- -0.35) | -0.54 (-1.75-0.67) | -0.03 (-0.90-0.84) |
| **Observation** | 0.19 (0.05-0.34) | - | - | - | - | - |
| **Palliative** | -0.48 (-0.99-0.03) | - | - | - | - | - |
| **Maintenance** |  |  |  |  |  |  |
| **Observation only** | - | - | - | - | reference | reference |
| **Maintenance** | - | - | - | - | -0.61 (-1.49-0.28) | 0.17 (-0.77-1.11) |
| **SCT** | - | - | - | - | - | - |
| **Maintenance + SCT** | - | - | - | - | - | - |
|  |  |  |  |  |  |  |
|  | **Time from Tx2 to death** | **Time from Tx2 to transform** | **Time from Tx2 to CR2** | **Time from CR2 to death** | **Time from CR2 to 2^nd^ relapse** |  |
| **Distribution** | Weibull | Weibull | Weibull | Weibull | Weibull |  |
| **Shape** | 1.23 (0.85-1.79) | 2.47 (1.82-3.34) | 0.75 (0.65-0.87) | 0.76 (0.51-1.14) | 0.87 (0.64-1.19) |  |
| **Intercept** | 11.16 (6.73-15.58) | 6.20 (3.69-8.71) | 4.23 (2.30-6.15) | 14.59 (8.91-20.27) | 7.87 (5.13-10.62) |  |
| **Age** | -0.06 (-0.11-0.002) | -0.003 (-0.04-0.03) | 0.02 (-0.004-0.05) | -0.09 (-0.16- -0.02) | -0.004 (-0.04-0.03) |  |
| **FLIPI** |  |  |  |  |  |  |
| **High** | reference | reference | reference | reference | reference |  |
| **Intermediate** | 0.04 (-1.39-1.47) | 0.06 (-0.88-0.99) | 0.20 (-0.60-1.00) | 1.11 (-1.08-3.30) | 0.73 (-0.49-1.94) |  |
| **Low** | -0.55 (-2.08-0.97) | 0.46 (-0.34-1.27) | -0.52 (-1.24-0.21) | -0.05 (-2.19-2.10) | 0.66 (-0.57-1.89) |  |
| **Not known** | 0.70 (-0.75-2.15) | 0.49 (-0.47-1.40) | -0.18 (-1.07-0.71) | -1.36 (-3.27-0.56) | -0.42 (-1.82-0.99) |  |
| **Treatment** |  |  |  |  |  |  |
| **CHOP** | -0.25 (-1.78-1.29) | 0.93 (0.21-1.65) | 0.52 (-0.33-1.37) | 0.68 (-1.75-3.11) | 0.88 (-0.44-2.20) |  |
| **CVP** | reference | reference | reference | reference | reference |  |
| **Chlorambucil** | -0.79 (-2.39-0.82) | 0.18 (-0.80-1.16) | -0.14 (-1.43-1.16) | 1.89 (-0.99-4.76) | 26.24 (-2777-2777) |  |
| **DHAP** | -0.14 (-2.25-1.98) | 0.25 (-0.47-0.98) | -0.21 (-1.14-0.72) | -2.40 (-5.49-0.69) | 0.89 (-0.95-2.73) |  |
| **Bendamustine** | -1.56 (-3.64-0.52) | 9.33 (-1274-1274) | -0.07 (-2.77-2.63) | 24.98 (-1275-1275) | 25.96 (-1018-1018) |  |
| **Other**† | -1.19 (-2.63-0.25) | 0.30 (-0.60-1.21) | -0.52 (-1.36-0.31) | -0.77 (-2.92-1.39) | 0.43 (-0.73-1.60) |  |
| **Radiotherapy** | -0.92 (-3.12-1.28) | 0.49 (-0.51-1.50) | -1.70 (-2.62- -0.77) | 0.56 (-1.61-2.73) | 0.15 (-1.08-1.37) |  |
| **Maintenance** |  |  |  |  |  |  |
| **Observation only** | - | - | - | reference | reference |  |
| **Maintenance** | - | - | - | 2.38 (0.13-4.62) | 0.31 (-0.64-1.26) |  |
| **SCT** | - | - | - | 1.46 (-1.59-4.51) | 0.39 (-1.68-2.46) |  |
| **Maintenance + SCT** | - | - | - | -1.08 (-5.00-2.85) | 0.16 (-2.58-2.89) |  |
|  |  |  |  |  |  |  |
|  | **Time from Tx3 to death** | **Time from Tx3 to transform** | **Time from Tx3 to CR3** | **Time from CR3 to death** | **Time from CR3 to 3^rd^ relapse** |  |
| **Distribution** | Weibull | Weibull | Weibull | Weibull | Weibull |  |
| **Shape** | 7.84 (3.18-19.34) | 7.94 (3.41-18.47) | 0.82 (0.58-1.16) | 2.49 (2.01-2.97) | 1.18 (0.70-2.00) |  |
| **Intercept** | 4.10 (0.66-7.54) | 6.05 (-0.74-12.84) | 6.23 (1.90-10.57) | 6.66 (1.15-12.12) | 7.03 (2.49-11.58) |  |
| **Age** | 0.02 (-0.02-0.07) | 0.004 (-0.08-0.09) | -0.01 (-0.07-0.05) | 0.02 (-0.06-0.09) | 0.04 (-0.03-0.11) |  |
| **FLIPI** |  |  |  |  |  |  |
| **High** | reference | reference | reference | reference | reference |  |
| **Intermediate** | -0.34 (-1.26-1.26) | 0.17 (-1.17-1.51) | -1.32 (-3.23-0.58) | 0.37 (-0.75-1.49) | 0.12 (-1.84-2.08) |  |
| **Low** | -0.44 (-1.91-1.03) | 2.67 (-0.97-6.31) | -1.37 (-3.06-0.31) | 1.29 (-1.02-3.60) | 1.99 (-0.16-4.13) |  |
| **Not known** | 2.80 (-3.98-9.58) | -0.36 (-1.74-1.02) | 0.02 (-1.62-1.65) | -0.29 (-2.37-1.79) | -1.54 (-3.78-0.71) |  |
| **Treatment** |  |  |  |  |  |  |
| **CHOP** | 3.57 (-1165-1165) | -0.51 (-1.87-0.86) | 0.72 (-0.68-2.11) | -0.77 (-6.69-5.15) | -2.48 (-5.21-0.25) |  |
| **CVP** | - | - | - | reference | reference |  |
| **Chlorambucil** | 0.12 (-0.28-0.52) | -0.40 (-1.42-0.63) | 1.44 (-1.22-4.10) | - | - |  |
| **DHAP** | 0.97 (-0.85-2.78) | 3.04 (-1182-1182) | -0.71 (-3.00-1.58) | -0.79 (-2.61-1.03) | -0.87 (-3.54-1.79) |  |
| **Bendamustine** | - | - | - | -2.18 (-5.22-0.86) | -4.55 (-7.36- -1.74) |  |
| **Other**† | reference | reference | reference | -0.36 (-3.76-3.04) | -2.31 (-5.00-0.38) |  |
| **Radiotherapy** | - | - | - | -0.77 (-3.99-2.45) | -3.01 (-5.59- -0.44) |  |
| **Maintenance** |  |  |  |  |  |  |
| **Observation only** | - | - | - | reference | reference |  |
| **Maintenance** | - | - | - | 0.33 (-2.17-2.83) | -0.34 (-2.09-1.41) |  |
| **SCT** | - | - | - | 0.78 (-2.63-4.19) | -0.14 (-2.47-2.18) |  |
| **Maintenance + SCT** | - | - | - | - | - |  |

†Including FC (Fludarabine and Cyclophosphamide), R-FC (Fludarabine, Cyclophosphamide and rituximab), FMD (Fludarabine, Mitoxantrone and Dexamethasone), R-FMD (Fludarabine, Mitoxantrone, Dexamethasone and rituximab), and R-Zevalin (Zevalin and Rituximab)

**Supplementary Table 6: Average health care utilisation**

| **Regimen** | **R-CHOP**  **n (95% CI)** | **R-CVP**  **n (95% CI)** | **R-Chlorambucil**  **n (95% CI)** | **R-DHAP**  **n (95% CI)** | **R-Bendamustine**  **n (95% CI)** | **Other**†  **n (95% CI)** | **Radiotherapy only**  **n (95% CI)** |
| --- | --- | --- | --- | --- | --- | --- | --- |
| **First line treatment** | 239 (238-239) | 622 (621-623) | 129 (129-129) | - | - | - | 281 (281-281) |
|  |  |  |  |  |  |  |  |
| Inpatient stay / day cases | 13.0 (10.2-15.7) | 9.4 (8.0-11.1) | 5.2 (2.8-7.6) | - | - | - | 2.4 (1.6-3.2) |
| Outpatient visits | 7.9 (6.3-9.5) | 11.5 (10.5-12.5) | 9.8 (8.3-11.2) | - | - | - | 6.2 (5.2-7.2) |
|  |  |  |  |  |  |  |  |
| **Second line treatment** | 128 (127-129) | 128 (127-129) | 48 (48-49) | 79 (79-80) | 8 (8-9) | 110 (110-111) | 70 (70-71) |
|  |  |  |  |  |  |  |  |
| Inpatient stay / day cases | 6.3 (3.3-9.3) | 3.1 (1.8-4.3) | 4.7 (2.4-7.1) | 4.1 (2.3-5.9) | 9.7 (7.1-12.3) | 4.4 (1.0-7.8) | 2.2 (0.8-3.6) |
| Outpatient visits | 11.4 (8.9-13.9) | 10.0 (7.5-12.5) | 9.0 (6.9-11.1) | 4.7 (3.0-6.4) | 11.3 (9.7-12.9) | 8.9 (6.6-11.1) | 6.3 (3.2-9.4) |
|  |  |  |  |  |  |  |  |
| **Third line treatment** | 22 (21-22) | 26 (26-27) | 4 (4-5) | 22 (21-22) | 4 (4-5) | 26 (26-27) | 17 (17-18) |
|  |  |  |  |  |  |  |  |
| Inpatient stay / day cases | 3.0 (1.2-4.8) | 4.2 (2.9-5.5) | 7.0 (5.7-8.3) | 13.3 (9.9-16.7) | 10.5 (7.6-12.4) | 11.2 (6.2-16.2) | 2.3 (1.4-3.2) |
| Outpatient visits | 8.4 (3.7-13.1) | 15.7 (11.4-19.9) | 14.0 (8.3-19.7) | 20 (12.6-27.4) | 19 (11.9-26.1) | 9.8 (4.7-14.9) | 4.5 (2.4-6.5) |
|  |  |  |  |  |  |  |  |

†Including FC (Fludarabine and Cyclophosphamide), R-FC (Fludarabine, Cyclophosphamide and rituximab), FMD (Fludarabine, Mitoxantrone and Dexamethasone), R-FMD (Fludarabine, Mitoxantrone, Dexamethasone and rituximab), and R-Zevalin (Zevalin and Rituximab)

**Supplementary Table 7: Comparison of simulated and relevant study results**

|  | **Country** | **Price year** | **Patient type** | **Model approach** | **Discount rate** | **Time horizon** | **Regimen** | **Mean Cost**  **(2016 UK pound *)** | **Mean time** |  | **Simulated cost** | **Simulated time** |
| --- | --- | --- | --- | --- | --- | --- | --- | --- | --- | --- | --- | --- |
| Carvalho et al. | Portugal | 2014 | Stage I, II, IIIa | Markov | 5% | 10 years | After 1^st^ line treatment  No maintenance  Maintenance | €36,708 (£30,179)  €44,173 (£36,317) | 6.28 yrs  6.98 yrs |  | £25,738  £24,714 | 7.1 yrs  8.0 yrs |
| Blommestein et al. | Netherlands | 2012 | Relapsed FL | Markov | 4% | 20 year | After 2^nd^ line treatment  No maintenance  Maintenance | €64,846 (£56,485)  €88,582 (£77,161) | 7.93 yrs  10.17 yrs |  | £51,002  £47,172 | 7.6 yrs  10.7 yrs |
| Kasteng et al. | Sweden | 2007 | Relapsed FL | Markov | 3% | 30 years | After 2^nd^ line treatment  No maintenance  Maintenance | €28,156 (£24,122)  €39,617 (£33,940) | 3.38 yrs  4.29 yrs |  | £54,670  £48,934 | 14.8 yrs  16.6 yrs |
| Deconinck et al. | France | 2006 | Relapsed FL | Markov | 3% | Lifetime | After 2^nd^ line treatment  No maintenance  Maintenance | €62,251 (£56,813)  €71,314 (£65,084) | 5.41 yrs  6.60 yrs |  | £61,350  £53,055 | 20.9 yrs  23.6 yrs |
|  |  |  |  |  |  |  |  |  |  |  |  |  |

* All currencies were inflated and converted to 2016 UK sterling pound.

**Supplementary Table 8: Cost results by subgroups**

|  | **Cost** | |  |
| --- | --- | --- | --- |
|  | **N** | **Mean (95% CI)** |  |
| Total | 1,860 | 18,705 (18,631-18,781) |  |
| Age (years) >60 |  |  |  |
| Yes | 1,222 (1,220-1223) | 16,381 (16,312-16,450) |  |
| No | 638 (636-649) | 25,168 (25,094-25242) |  |
| Follicular Lymphoma International Prognostic Index (FLIPI) |  |  |  |
| Low (0-1 risk factor) | 483 (481-484) | 14,612 (14,554-14,671) |  |
| Intermediate (2 risk factors) | 432 (170-173) | 20,102 (20,022-20,183) |  |
| High (≥ 3 risk factors) | 684 (271-274) | 22,742 (22,652-22,834) |  |
| Not done | 261 (103-105) | 13,461 (13,408-13,516) |  |
| First line treatment |  |  |  |
| Chemotherapy | 839 (838-841) | 26,530 (26,425-26,635) |  |
| Radiotherapy | 94 (92-95) | 16,933 (16,852-17,014) |  |
| Radiotherapy for Stage IA | 159 (157-160) | 17,956 (17,884-18,028) |  |
| Observation | 731 (729-730) | 12,431 (12,259-12,603) |  |
| Not treated | 37 (36-38) | 6165 (6,093-6,237) |  |


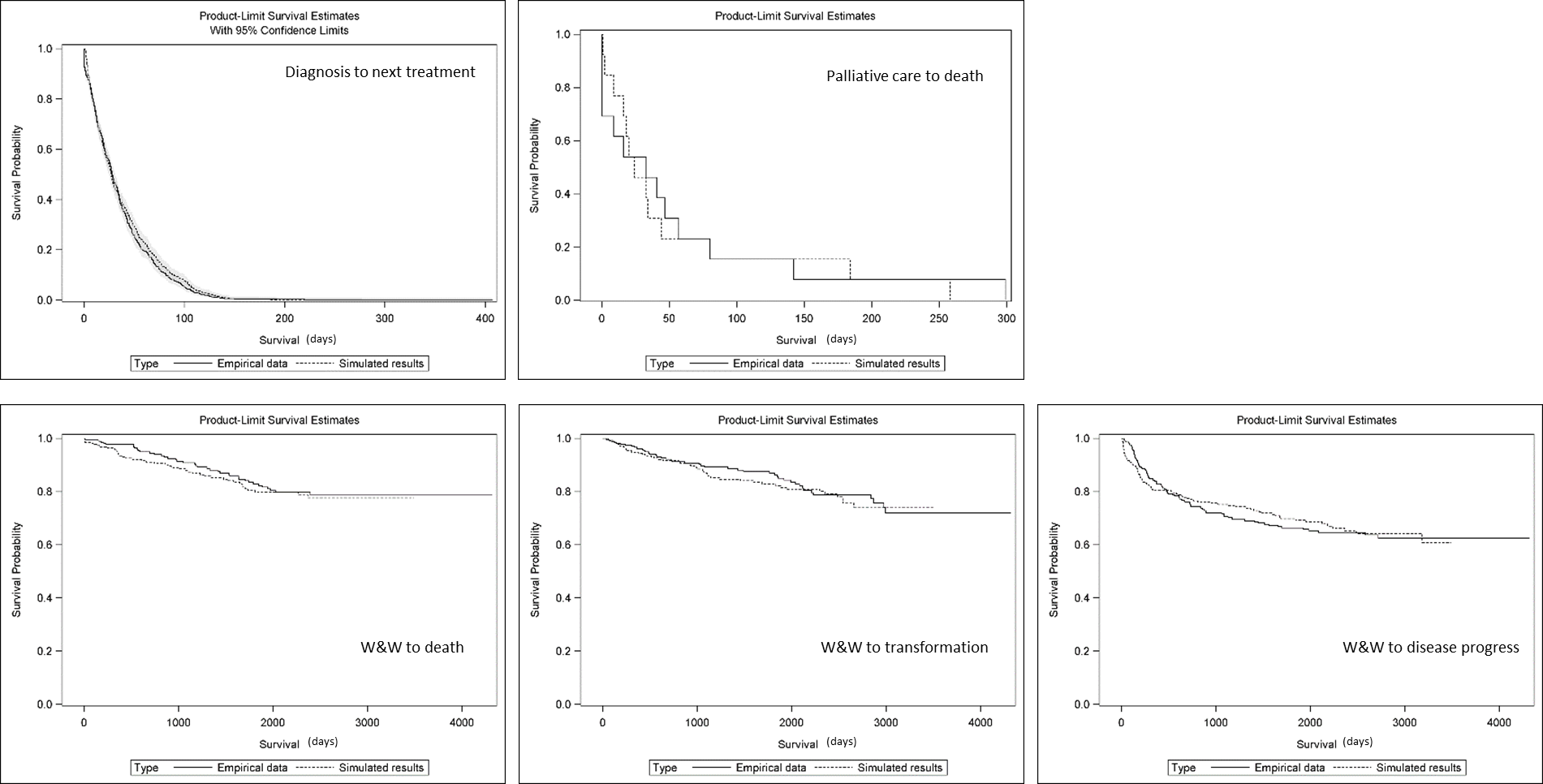


**Supplementary Figure 1a: Kaplan Meier curves of both observed and simulated data**


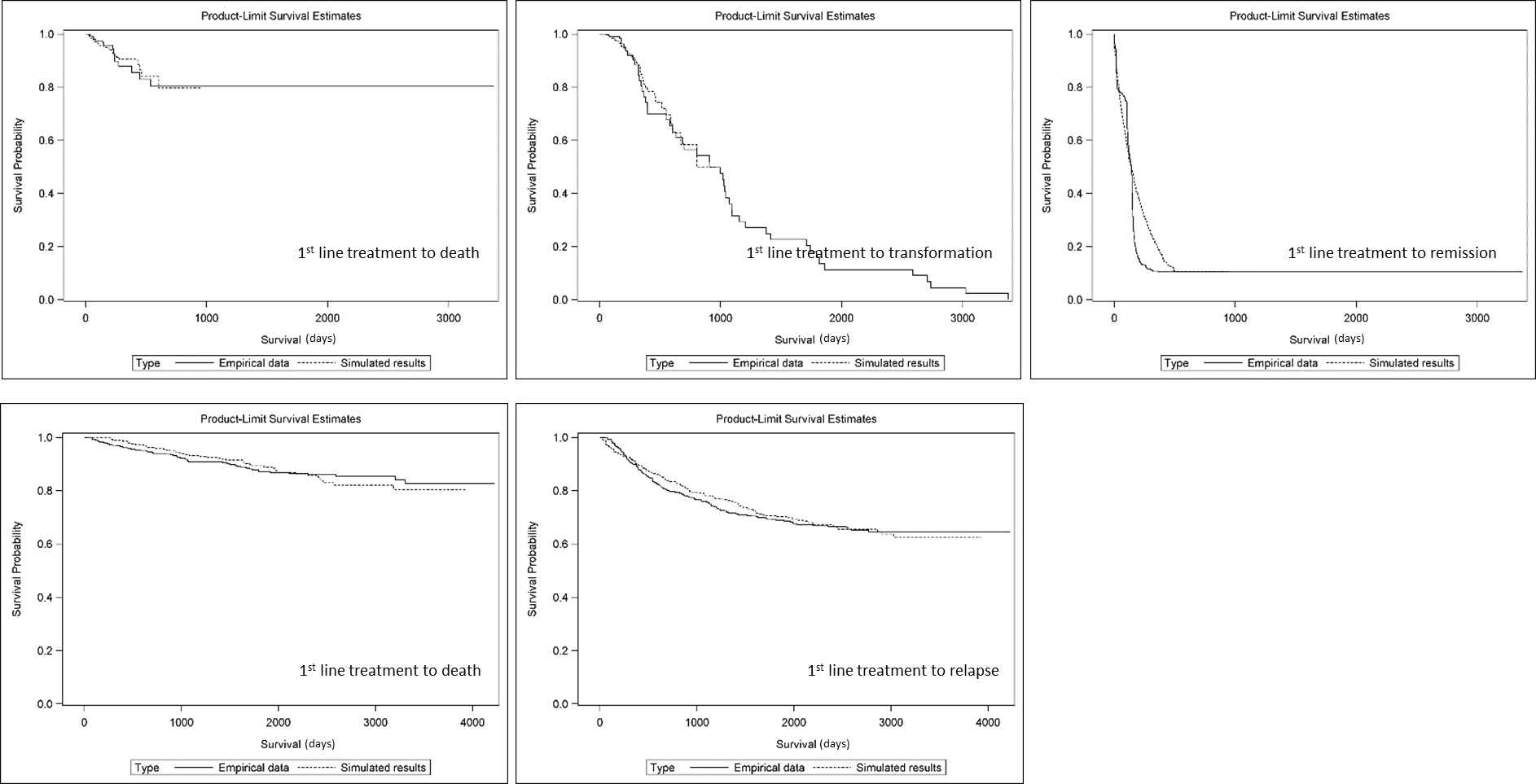


**Supplementary Figure 1b: Kaplan Meier curves of both observed and simulated data**


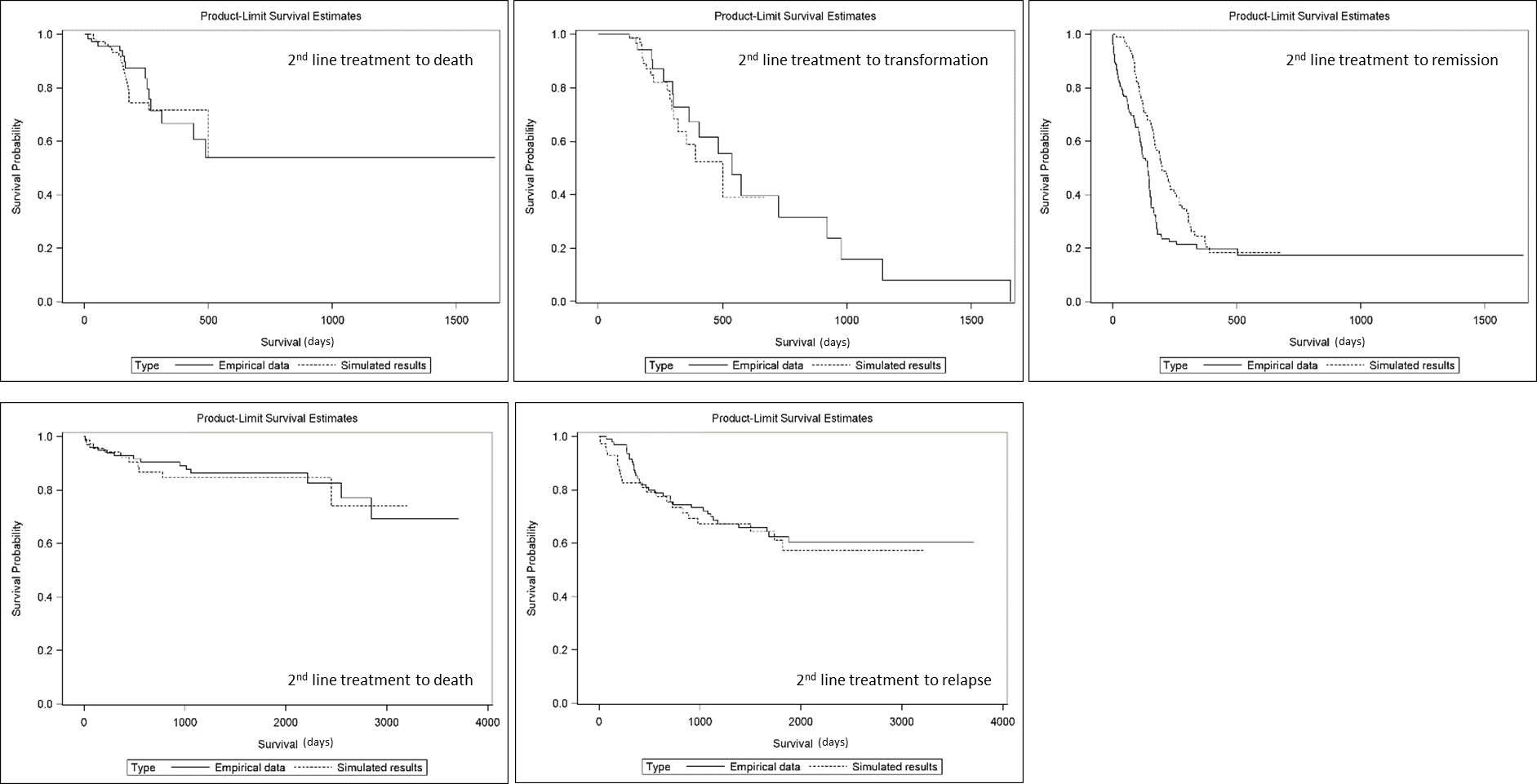


**Supplementary Figure 1c: Kaplan Meier curves of both observed and simulated data**


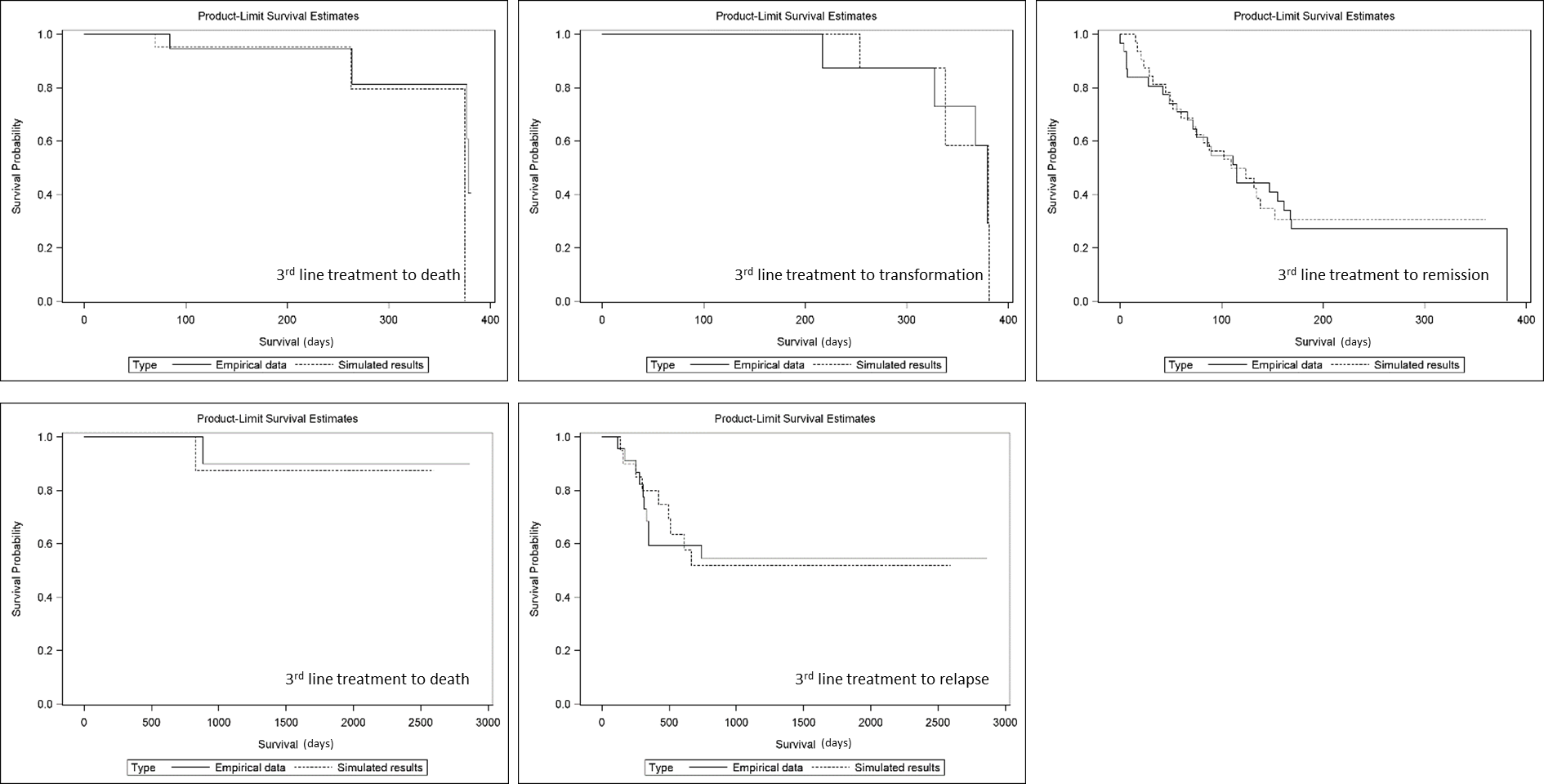


**Supplementary Figure 1d: Kaplan Meier curves of both observed and simulated data**


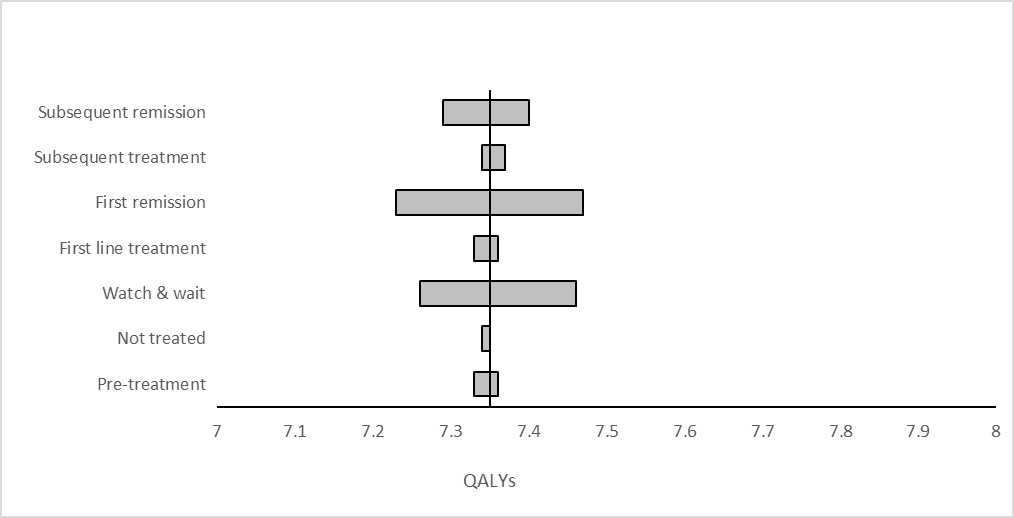


Utility

0.70-0.96

0.41-0.59

0.82-0.89

0.79-0.87

0.85-0.91

0.58-0.66

0.75-0.83

Lower 95% CI-Upper 95% CI

**Supplementary Figure 2: One way sensitivity analysis on utility values**
